# Associated thromboembolic events to the post COVID syndrome: a systematic review and meta-analysis

**DOI:** 10.3389/fcvm.2026.1742868

**Published:** 2026-06-11

**Authors:** Jesús Endara-Mina, Cristopher-Josue Escudero, Cesar Intriago, Lisseth Coloma-Ramirez, Wilson Cabrera, Rosa Gonzaga, Sebastian Fuentes, Dario Chicaiza, Carlos Carvajal, Rafael Lopez-Carrera, Belen Baño-Jimenez, Jordy Ruilova, Erick Atahualpa, Josue Aldaz, Mayuri Quishpe, Katherine Simbaña-Rivera

**Affiliations:** 1 Facultad de Ciencias Jurídicas y Políticas, Universidad Técnica Particular de Loja (UTPL), Loja, Ecuador; 2 Coordinación de Docencia e Investigación, Hospital Provincial General Pablo Arturo Suárez, Quito, Pichincha, Ecuador; 3 Scientific Association of Medical Students, Universidad Central del Ecuador (UCE), Quito, Ecuador; 4 Facultad de Ciencias Médicas, Universidad Central del Ecuador (UCE), Quito, Ecuador; 5 Facultad de Salud y Bienestar, Pontificia Universidad Católica del Ecuador (PUCE), Quito, Ecuador; 6 Facultad de Salud Pública, Escuela Politécnica de Chimborazo (ESPOCH), Riobamba, Ecuador; 7 Research Institute of Biomedical and Health Sciences (IUIBS), Universidad de Las Palmas de Gran Canaria (ULPGC), Las Palmas de Gran Canaria, Spain

**Keywords:** deep vein thrombosis, hemostasis, long-haul COVID, post COVID-19 syndrome, thromboembolism

## Abstract

**Background:**

The COVID-19 pandemic has imposed a substantial worldwide health burden; a fraction of people develops post-COVID syndrome, in which symptoms persist long after the initial phase of infection. Although these individuals may be more susceptible to developing thromboembolic events, the extent and significance of this link remain uncertain. This systematic review and meta-analysis sought to explore the prevalence of thromboembolic events in people with post-COVID syndrome, therefore addressing knowledge gaps and providing critical information for therapeutic management.

**Methods:**

Following PRISMA principles, a thorough search across numerous databases—including Medline, Web of Science, Scopus, Dimensions, the Virtual Health Library, the British Library, and Google Scholars—was performed. Analytical cross-sectional, case-control, and cohort studies on individuals with post-COVID syndrome were considered eligible research. Meta-analysis was performed using Review Manager 5.4.1, with a random-effects model providing hazard ratios (HR) and 95% confidence intervals (CI) for specific thromboembolic events.

**Results:**

The initial database search yielded 1,617 publications, 1,021 of which passed title and abstract screening after duplicates were removed. Following a full-text analysis of 83 publications, 20 met the inclusion criteria, with 5 included in the quantitative synthesis and 15 in the qualitative synthesis.

**Conclusion:**

Emphasizing ramifications for clinical therapy, particularly among vascular surgeons, this thorough review and meta-analysis exposes a significant thromboembolic risk among patients with post-COVID syndrome. The outcomes highlight the need for early detection, proactive monitoring, and tailored preventive treatments, including anticoagulant drugs.

**Systematic Review Registration:**

https://www.crd.york.ac.uk/PROSPERO/view/CRD42023441556, PROSPERO CRD42023441556.

## 1 Introduction

The coronavirus disease 2019 (COVID-19) pandemic has resulted in substantial global morbidity, mortality, and long-term health consequences, extending beyond the acute phase of severe acute respiratory syndrome coronavirus 2 (SARS-CoV-2) infection (1, 2). While the acute manifestations of COVID-19 have been extensively characterized, increasing attention has been directed toward a subset of individuals who experience persistent or newly emerging symptoms weeks to months after the initial infection, a condition commonly referred to as post-COVID-19 syndrome (3–5).

Post-COVID-19 syndrome is characterized by a heterogeneous constellation of symptoms—including fatigue, dyspnea, chest pain, cognitive disturbances, and musculoskeletal complaints—that may persist or newly emerge beyond the acute phase of infection, with variability in duration across studies (6, 7). This condition affects individuals across the spectrum of initial disease severity, including those with mild or asymptomatic infections, and represents a growing challenge for healthcare systems due to its prolonged clinical course and impact on quality of life (8–10).

One of the most clinically relevant concerns associated with post-COVID-19 syndrome is the potential persistence of a prothrombotic state. During acute SARS-CoV-2 infection, a complex interplay of systemic inflammation, endothelial dysfunction, platelet activation, and coagulation pathway dysregulation contributes to an increased risk of venous and arterial thromboembolic events (11, 12). Elevated levels of inflammatory markers, endothelial injury, and sustained coagulation abnormalities have been documented during the acute phase, raising concern that these mechanisms may persist beyond viral clearance (11). This inflammatory-coagulopathic milieu increases the risk of deep vein thrombosis (DVT), pulmonary embolism (PE), and arterial thromboembolism (ATE), with clinical manifestations that may include myocardial infarction (MI), ischemic stroke (IS), and peripheral arterial occlusion (PAO) (13, 14). These complications can arise during the acute phase but may also persist or emerge weeks to months after resolution of the primary infection, characterizing post-COVID syndrome.

A comprehensive synthesis of the available evidence is therefore required to better characterize the association between post-COVID-19 syndrome and thromboembolic events. Clarifying this relationship is essential to inform clinical surveillance strategies, guide future research, and identify populations that may benefit from closer monitoring. Accordingly, this systematic review and meta-analysis aims to synthesize observational evidence on the occurrence of venous and arterial thromboembolic events in individuals with post-COVID-19 syndrome and to quantify the associated risk where data permit.

## 2 Methods

This study was conducted as a systematic review and meta-analysis of observational studies, following the Preferred Reporting Items for Systematic Reviews and Meta-Analyses (PRISMA) 2020 guidelines (15). The review protocol was prospectively registered in the International Prospective Register of Systematic Reviews (PROSPERO; CRD42023441556). No major deviations from the registered protocol were performed after study selection.

### 2.1 Research aim

The research question was formulated using the Population–Exposure–Comparison–Outcome (PECO) framework. The population of interest included individuals diagnosed with post-COVID-19 syndrome. For the purposes of this review, we examined post-acute and chronic post-COVID-19 syndrome, encompassing outcomes occurring beyond the acute phase of confirmed severe acute respiratory syndrome coronavirus 2 (SARS-CoV-2) infection. Given heterogeneity across observational studies, an operational approach was adopted, prioritizing medium- (approximately 3–12 weeks) and long-term follow-up periods in line with contemporary conceptualizations of post-acute sequelae of COVID-19 (16). The exposure of interest was post-COVID-19 syndrome. Comparator groups, when available, consisted of individuals without post-COVID-19 syndrome. Outcomes of interest included venous and arterial thromboembolic events, such as deep vein thrombosis, pulmonary embolism, ischemic stroke, myocardial infarction, and other clinically relevant thrombotic outcomes.

Post-COVID-19 syndrome is characterized by a heterogeneous constellation of symptoms that may persist or newly emerge beyond the acute phase of infection. This condition is also commonly referred to as “long COVID” in the literature, although terminology and case definitions vary across studies.

### 2.2 Eligibility criteria

Observational studies, including cohort, case-control, and analytical cross-sectional designs, were eligible for inclusion if they evaluated thromboembolic events in individuals with post-COVID-19 syndrome. Studies involving pediatric populations or pregnant women were excluded. Only studies published in English or Spanish with full-text availability were considered. Case reports, case series, narrative reviews, editorials, conference abstracts, preprints, no randomized or quasi-experimental studies were included in the quantitative synthesis and studies with insufficient methodological detail were excluded. Studies assessed as having a critical risk of bias were excluded from the quantitative synthesis (Table 1).

**Table 1:** Inclusion and exclusion criteria.

Domain	Inclusion	Exclusion
Population	Individuals diagnosed with post–COVID-19 syndrome	Pregnant women; pediatric populations
Language	English or Spanish	Other languages
Study design	Observational studies: cohort, case–control, and analytical cross-sectional studies	Case reports, case series, narrative reviews, editorials, conference abstracts, protocols, and preprints
Risk of bias	Studies with non-critical risk of bias based on the Newcastle–Ottawa Scale.	Studies judged as having critical risk of bias on the Newcastle–Ottawa Scale.
Publication date	No restrictions	Not applicable
Access	Full-text available	Studies without full-text access

### 2.3 Information sources and search

A comprehensive literature search was performed in PubMed/MEDLINE, Web of Science, Scopus, Dimensions, the Virtual Health Library, the British Library, and Google Scholar. The final search was conducted on December 31, 2023, without restrictions on publication date. Search strategies combined Medical Subject Headings (MeSH) terms and free-text keywords related to COVID-19, post-COVID-19 syndrome, and thromboembolic events. The complete search strategy for each database is provided in the Supplementary Material.

### 2.4 Study selection and data extraction

All retrieved records were imported into Rayyan software for deduplication and screening (17). Two reviewers independently screened titles and abstracts for eligibility, followed by full-text assessment of potentially relevant articles. Discrepancies were resolved through discussion, and when necessary, by consultation with a third reviewer. The study selection process is summarized using a PRISMA flow diagram.

Data extraction was performed independently by two reviewers using a standardized extraction form adapted from the Cochrane Consumers and Communication Review Group template. Extracted data included study characteristics (authors, year, country, study design), population characteristics, sample size, follow-up duration, definitions of post-COVID-19 syndrome, thromboembolic outcomes assessed, effect estimates, and key findings. Any disagreements were resolved by consensus.

### 2.5 Risk of bias assessment

The risk of bias and methodological quality assessment was conducted independently by two reviewers using the Newcastle–Ottawa Scale (NOS) for post-exposure studies (18). Given the nature of the included articles—studies evaluating outcomes occurring after exposure to and infection with COVID-19—this scale addresses the most relevant methodological criteria for their evaluation. The NOS allows the concurrent assessment of methodological quality and risk of bias by examining study selection, comparability between groups, and outcome ascertainment through a star-based grading system. Quality categories were classified as low, low–moderate, moderate, moderate–high, and high, with higher scores indicating greater methodological quality and a correspondingly lower risk of bias (18).

### 2.6 Data synthesis and statistical analysis

A systematic review of observational studies was conducted. Five studies were included in the meta-analysis, and 15 studies were retained for qualitative synthesis. Statistical analyses were performed using Review Manager version 5.4.1 (RevMan). The summary measure was the hazard ratio (HR), as it accounts for both event occurrence and time-to-event, enabling a more precise assessment of relative risk in medium- and long-term follow-up. Studies were grouped by outcome to generate pooled HRs for each subgroup.

Pooled estimates were calculated using the logarithm of the HR and corresponding 95% confidence intervals (CIs) under a random-effects model. Results were reported as HRs with 95% CIs. Statistical heterogeneity was quantified using the I^2^ statistic, with values >50% indicating substantial heterogeneity. Statistical significance was defined as a two-sided *p*-value <0.05. The random-effects model implemented in RevMan is based on the DerSimonian and Laird method, which accounts for variability both within and between studies.

### 2.7 Grading of evidence and recommendations

The certainty of evidence for each outcome was evaluated using the Grading of Recommendations, Assessment, Development and Evaluations (GRADE) approach across the following domains: risk of bias, inconsistency, indirectness, imprecision, and publication bias (19). Because all studies included in the quantitative synthesis demonstrated high methodological quality according to the NOS, no downgrading was performed for risk of bias. Certainty was downgraded primarily due to inconsistency across study populations and follow-up durations, as well as imprecision related to variability in effect estimates and confidence intervals. No major concerns were identified regarding indirectness, while publication bias could not be formally assessed due to the limited number of studies included in each meta-analysis.

## 3 Results

**Figure 1:**
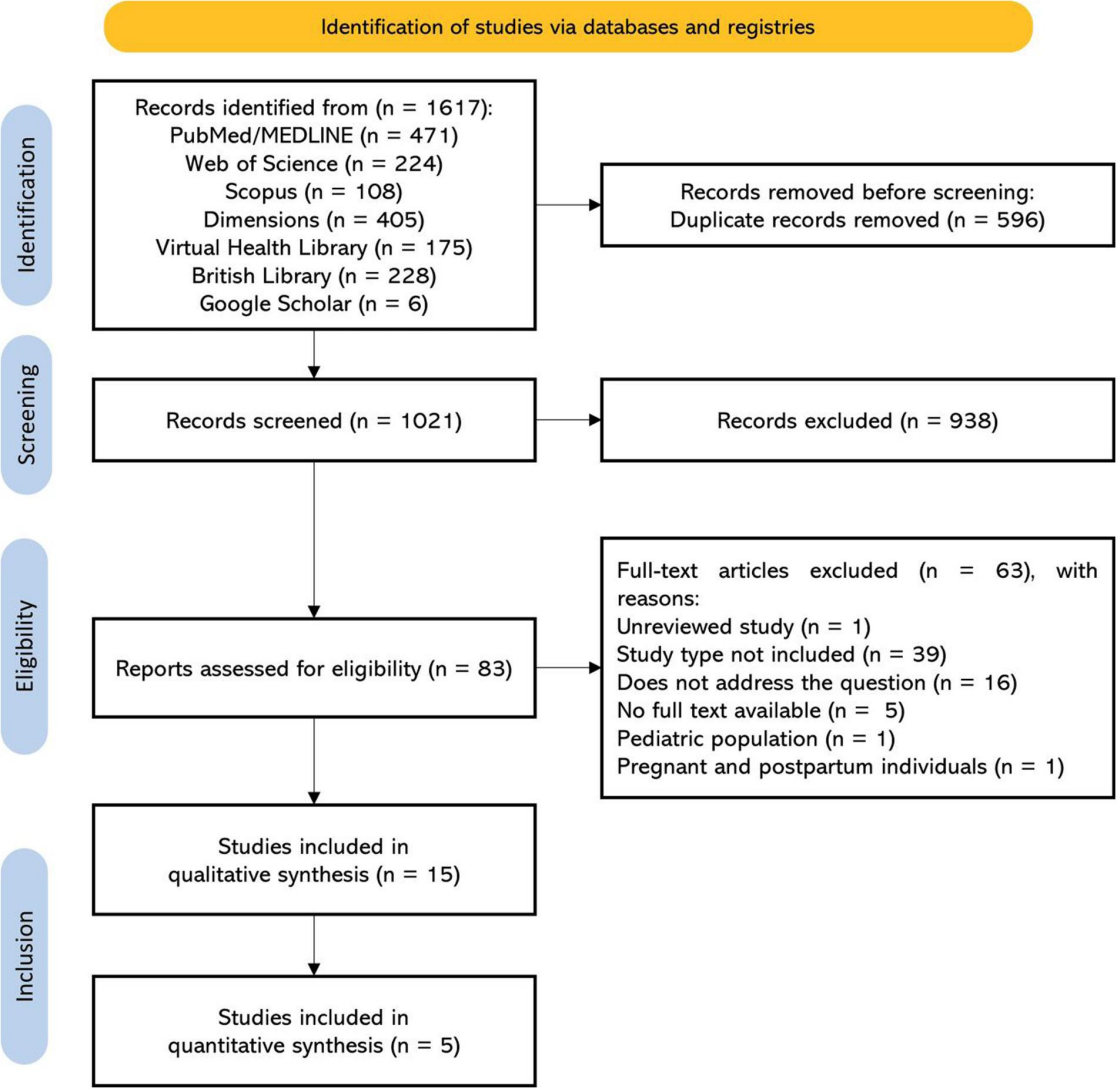
PRISMA flow diagram of study screening and selection. PRISMA: Preferred Reporting Items for Systematic Reviews and Meta-Analyses.

The database search identified a total of 1,617 records. After removal of duplicates, 1,021 studies were screened by title and abstract. Of these, 83 articles underwent full-text review, and 20 studies met the inclusion criteria (Figure 1). Five studies were eligible for quantitative synthesis, while 15 were included in the qualitative synthesis. The primary reasons for exclusion were inappropriate study design, lack of relevant outcomes, or insufficient methodological detail.

### 3.1 Characteristics of the studies

A total of 20 studies met the inclusion criteria and were incorporated into the systematic review. Five population-based cohort studies were included in the quantitative synthesis, and 15 observational or mechanistic studies were included in the qualitative synthesis (Tables 2, 3). The selected studies were published between 2020 and 2023 and represented a wide geographical distribution across Europe, North America, South America, Asia, and Africa.

**Table 2:** Characteristics of the studies included in the quantitative synthesis.

Author	Year	Study design	Country	Age (mean ± SD)	Size	Female (%)
Lam, et al. (13)	2023	Cohort	China	53.6 ± 17.8	8516716	55.8
			UK	65.0 ± 8.5		
Xu, et al. (14)	2022	Cohort	USA	61.4 ± 15.6	11652484	9.5
Junqing Xie, et al. (20)	2022	Cohort	UK	64.3 ± 8.3	448787	57.7
Ortega-Paz, et al. (21)	2022	Cohort	Spain	63.1 ± 17.3	4427	46.1
Xie, et al. (22)	2022	Cohort	USA	61.4 ± 15.6	11650818	9.5

SD, standard deviation; UK, United Kingdom; USA, United States of America.

**Table 3:** Characteristics of the studies included in the qualitative synthesis.

Author	Year	Study design	Country	Age (mean ± SD or median + range)	Size	Female (%)
Lund, et al. (23)	2021	Cohort	Denmark	43 (30 to 56)	89877	63.5
Ogoina, et al. (24)	2021	Cohort	Nigeria	49 (34 to 52)	30	33.3
Chhabra, et al. (25)	2021	Cohort	India	51 (6.4) ^a^	40	15
				44 (6.8) ^b^		
Voss, et al. (26)	2023	Cohort	Belgium, Estonia, France, Germany, Japan, the Netherlands, Serbia, Spain, Turkey, UK, and USA	39 (24 to 72)	945520607	47.4 to 56.1
Giannis, et al. (27)	2021	Cohort	USA	61 (17.5)	4906	46.3
Rezel-Potts, et al. (28)	2022	Cohort	UK	35 (22 to 50)	557300	56
Ranucci, et al. (29)	2023	Cohort	Italy	63 (13.1)	102	34.3
Donnachie, et al. (30)	2022	Cohort	Germany	42 (21.0) ^c^	1114228	54.1
Roberto, et al. (31)	2023	Cross - sectional	Brazil	59 (14.0)	480	32.5
de Miranda, et al. (32)	2022	Cohort	Brazil	50 (15.7)	646	53.9
Kamal, et al. (33)	2021	Cross - sectional	Egypt	32 (8.5)	287	64.1
Constantinescu-Bercu, et al. (34)	2023	Cross - sectional	UK	46 (28 to 70)	21	81
Karlović, et al. (35)	2023	Cross - sectional	Bosnia and Herzegovina	71 (5.2) ^d^	53	30.2
Kartsios, et al. (36)	2020	Cohort	UK	68 (29 to 91)	38	47
Bhandari, et al. (37)	2023	Cohort	India	(30 to 70)	3220	38.5

SD, standard deviation; UK, United Kingdom; USA United States of America.

^a^ Male.

^b^ Female.

^c^ Population values with confirmed Covid-19 diagnosis.

^d^ The average age of post COVID-9 patients.

The majority of studies were multinational or population-based, and both hospitalized and non-hospitalized post-COVID-19 patients were evaluated. Participant ages ranged from 30 to 70 years, with a female representation between 9.5% and 63.5%. The main outcomes assessed included DVT, PE, IS, ATE, and major adverse cardiovascular events (MACE).

### 3.2 Risk of bias assessment

Risk of bias was assessed using the NOS. Among the 20 included articles, 9 demonstrated high methodological quality with a low risk of bias. One article showed moderately high methodological quality with a comparable risk of bias. Nine studies were rated as having moderate quality, and only one study showed low-to-moderate methodological quality, with a corresponding risk of bias. Table 4 summarizes the NOS assessment. All studies included in the meta-analysis achieved high methodological quality and, consequently, a low risk of bias. Overall, GRADE assessments ranged from moderate to low certainty across outcomes, primarily reflecting heterogeneity in follow-up periods and variability in reported effect estimates (Supplementary Material).

**Table 4:** Methodological quality assessment of the included studies using the Newcastle–Ottawa scale (NOS).

Author (year)	Selection	Comparability	Outcome ascertainment	Total (0–9)	Quality
Lam ICH, et al. (2023) (13)	★★★★	★	★★★	8	High
Xu E, et al. (2022) (14)	★★★★	★	★★★	8	High
Xie J, et al. (2022) (20)	★★★★	★	★★★	8	High
Ortega-Paz L, et al. (2022) (21)	★★★★	★	★★★	8	High
Xie Y, et al. (2022) (22)	★★★★	★	★★★	8	High
Lund LC, et al. (2021) (23)	★★★★	★	★★★	8	High
Ogoina D, et al. (2021) (24)	★★★	-	★★★	6	Moderate
Chhabra A. et al. (2021) (25)	★★★	-	★★★	6	Moderate
Voss EA, et al. (2023) (26)	★★★★	★	★★★	8	High
Giannis D, et al. (2021) (27)	★★★	★	★★★	7	Moderate-High
Rezel-Potts E, et al. (2022) (28)	★★★★	★	★★★	8	High
Ranucci M, et al. (2023) (29)	★★★	-	★★★	6	Moderate
Donnachie E, et al. (2022) (30)	★★★★	★	★★★	8	High
Roberto K-F, et al. (2023) (31)	★★★	-	★★★	6	Moderate
De Miranda DAP, et al. (2022) (32)	★★★	-	★★★	6	Moderate
Kamal M, et al. (2021) (33)	★★	-	★★★	5	Moderate
Constantinescu-Bercu A, et al. (2023) (34)	★★★	-	★★★	6	Moderate
Karlovic K, et al. (2022) (35)	★★	-	★★★	5	Moderate
Kartsios C, et al. (2021) (36)	★★	-	★★★	5	Moderate
Bhandari S, et al. (2023) (37)	★★	-	★★	4	Low-Moderate

The NOS evaluates three principal domains: selection of the study sample and its representativeness, comparability of cohorts, and outcome ascertainment. Overall, high methodological quality was identified in nine of the included studies. One study demonstrated moderate-to-high quality, nine studies were classified as having moderate methodological quality, and one study showed low-to-moderate quality. Across studies, the domain most frequently associated with potential bias was comparability, reflecting limited adjustment for confounding variables in several analyses. In contrast, outcome ascertainment was generally robust, representing the strongest methodological domain among the included investigations.

### 3.3 Synthesis of findings

The individual synthesis of the included studies is presented in Table 5, which provides a detailed description of the main methodological, population, and clinical characteristics of each investigation, as well as the outcomes related to thromboembolic events associated with post-COVID syndrome.

**Table 5:** Summary of included studies.

Ref.	Objective	Sample/Follow-up Time	Observed event	Results	Analysis
(29)	To evaluate the long-term residual effects of COVID-19-associated coagulopathy through conventional and viscoelastic laboratory tests, and to analyze the relationship between persistent hemostatic alterations and prolonged physical and neuropsychological symptoms in previously hospitalized patients with SARS-CoV-2 infection.	Sample: 102 patients. Follow-up time: median duration of 17 months	Persistent procoagulopathy: Presence of at least one of the following: fibrinogen > 400 mg/dL, D-dimer > 500 ng/mL, platelet count > 450,000/µL, antithrombin activity < 70%, or EXTEM maximum lysis (ML) < 2%. Persistence of physical symptomatology: Fatigue (49%), dyspnea (43.1%), cough (12.7%), fever (2%) Presence of neurocognitive or neuropsychological symptomatology Anxiety, depression, memory impairment, and brain fog Decreased fibrinolytic capacity EXtest ML < 5.9%, INtest ML < 5.5%, prolonged APTT test Persistent dyspnea Predominant symptom in respiratory long COVID Advanced age (>50 years) Predisposing factor for endothelial dysfunction and coagulopathy. Severity of acute COVID-19 Mild, moderate, or severe (based on oxygen and/or ventilatory support requirements)	− 38 patients (37.3%) at follow-up (17 months) —75% at 3 months, 50% at 6 months, 30% at 12–18 months – R^2^ = 0.415, *p* = 0.001 (cubic regression of time vs. symptom persistence) 60/102 (58.8%) patients RR = 2.8 (95% CI: 1.17–6.7), *p* = 0.019 44/102 (43.1%) patients Not significantly associated with procoagulopathy – EXTEM ML in PPS: 4.5% ± 2.3 vs. 5.9% ± 2.6 – INTEM ML in PPS: 4.1% ± 2.3 vs. 5.5% ± 2.5 – APTEM lysis time in PPS: 227 s vs. 209 s 44/102 patients (43.1%) Ntest ML: 3.9% vs. 5.3% (*p* = 0.004) EXtest ML: 4.4% vs. 5.6% (*p* = 0.017) Progressive increase up to 90% in patients >80 years *p* *=* *0.001* (age-stratified analysis) Greater persistence of coagulopathy in severe cases *p* *=* *0.05* (statistically significant)	The study demonstrates that a procoagulant state may persist for up to 18 months following hospital discharge in a considerable proportion of post-COVID patients. This hemostatic pattern is characterized by increased clot firmness (MCF), reduced fibrinolytic capacity (ML), and elevated D-dimer levels in approximately 30% of the individuals assessed. These findings suggest the presence of an ongoing process of microthrombi formation and incomplete degradation within the vascular system. Furthermore, a significant association was identified between the presence of physical symptoms—particularly dyspnea—and impaired fibrinolysis, which may help explain some of the underlying pathophysiological mechanisms of long COVID syndrome.
(28)	To evaluate the incidence of new-onset cardiovascular disease (CVD) and diabetes mellitus (DM) up to 12 months after COVID-19 infection, compared with matched uninfected controls.	Sample: 428,650 patients with COVID-19 matched to 428,650 controls Follow-up time: 12 months	Diabetes mellitus (DM) Total cardiovascular disease (CVD) Acute CVD subgroup	– Acute phase (0–4 weeks): RR 1.81 (95% CI: 1.51–2.19) – Post-acute phase (5–12 weeks): RR 1.27 (95% CI: 1.11–1.46) – Long COVID phase (13–52 weeks): RR 1.07 (95% CI: 0.99–1.16) Total CVD: – Acute phase: RR 5.82 (95% CI: 4.82–7.03) – Post-acute phase: RR 1.49 (95% CI: 1.28–1.73) – Long COVID phase: RR 0.80 (95% CI: 0.73–0.88) – Pulmonary embolism: RR 11.51 – Arrhythmias: RR 6.44 – Venous thrombosis: RR 5.43 – Myocardial infarction/ischemic heart disease: RR 2.01	During the first 4 weeks post-COVID, a marked increase in cardiovascular risk was observed, particularly due to pulmonary embolism and arrhythmias. This risk progressively declined over the course of the year. For diabetes mellitus, the risk remained elevated up to 12 weeks, with slight persistence thereafter. COVID-19 infection functions as a transient precipitating factor for cardiometabolic events.
(34)	To evaluate the persistent prothrombotic state in patients with post-COVID syndrome using flow-based thrombogenicity assays, thrombin generation tests, and hemostatic markers such as von Willebrand factor and ADAMTS13.	Sample: 21 symptomatic PCS patients ≥13 months post-infection and 45 healthy age-matched controls Follow-up time: Median of 23 months	Increased platelet adhesion Increased thrombus formation (area and length) Increased thrombin generation Elevated VWF(Ag):ADAMTS13 ratio Marginally elevated *α*2-antiplasmin and fibrinogen	Significantly greater adhesion in PCS patients compared to controls Trombos significativamente más largos en PCS (*p* < 0.0001) 10/18 patients (55.5%) with elevated AUC (>2945 nM)Significant at the cohort level 8/21 patients (38%) with a ratio ≥ 1.5 Abnormal ratio associated with increased thrombogenicity 17/19 patients (89.5%) within the normal range	A persistent prothrombotic state in PCS is confirmed, mediated by increased VWF levels and enhanced thrombin generation. The alterations observed under dynamic flow conditions simulate a real vascular environment, reinforcing the clinical relevance of the findings. Persistent dysfunction of the VWF/ADAMTS13 axis may help explain symptoms such as fatigue or dyspnea due to microthrombosis.
(22)	To estimate the 12-month risk and burden of incident cardiovascular diseases following COVID-19	Sample: COVID-19 group: 153,760 individuals who survived at least 30 days after confirmed diagnosis Contemporary control group: 5,637,647 Veterans Health Administration (VHA) users with no evidence of SARS-CoV-2 infection Follow-up time: 12 months	Cerebrovascular disorders Arrhythmias Inflammatory heart diseases Ischemic heart disease Other cardiac disorders Thromboembolic disorders	Stroke: HR: 1.52 (95% CI: 1.43–1.62); Burden: 4.03 per 1,000 persons Transient ischemic attack (TIA): HR: 1.49 (95% CI: 1.37–1.62); Burden: 1.84 per 1,000 Composite stroke+TIA: HR: 1.53 (95% CI: 1.45–1.61); Burden: 5.48 per 1,000 Atrial fibrillation: HR: 1.71 (95% CI: 1.64–1.79); Burden: 10.74 per 1,000 Sinus tachycardia: HR: 1.84 (95% CI: 1.74–1.95); Burden: 5.78 per 1,000 Sinus bradycardia: HR: 1.53 (95% CI: 1.45–1.62); Burden: 4.62 per 1,000 Ventricular arrhythmias: HR: 1.84 (95% CI: 1.72–1.98); Burden: 4.18 per 1,000 Atrial flutter: HR: 1.80 (95% CI: 1.66–1.96); Burden: 3.10 per 1,000 Pericarditis: HR: 1.85 (95% CI: 1.61–2.13); Burden: 0.98 per 1,000 Myocarditis: HR: 5.38 (95% CI: 3.80–7.59); Burden: 0.31 per 1,000 Acute coronary disease: HR: 1.72 (95% CI: 1.56–1.90); Burden: 5.35 per 1,000 Myocardial infarction: HR: 1.63 (95% CI: 1.51–1.75); Burden: 2.91 per 1,000 Ischemic cardiomyopathy: HR: 1.75 (95% CI: 1.44–2.13); Burden: 2.34 per 1,000 Angina: HR: 1.52 (95% CI: 1.42–1.64); Burden: 2.50 per 1,000 Heart failure: HR: 1.72 (95% CI: 1.65–1.80); Burden: 11.61 per 1,000 Non-ischemic cardiomyopathy: HR: 1.62 (95% CI: 1.52–1.73); Burden: 3.56 per 1,000 Cardiac arrest: HR: 2.45 (95% CI: 2.08–2.89); Burden: 0.71 per 1,000 Cardiogenic shock: HR: 2.43 (95% CI: 1.86–3.16); Burden: 0.51 per 1,000 Pulmonary embolism (PE): HR: 2.93 (95% CI: 2.73–3.15); Burden: 5.47 per 1,000 Deep vein thrombosis (DVT): HR: 2.09 (95% CI: 1.94–2.24); Burden: 4.18 per 1,000 Superficial vein thrombosis: HR: 1.95 (95% CI: 1.80–2.12); Burden: 2.61 per 1,000	The study demonstrated a clear and significant association between prior SARS-CoV-2 infection and an increased risk of developing multiple cardiovascular diseases during the post-acute phase (beyond 30 days after infection), even among non-hospitalized patients. The most prevalent incident cardiovascular events following COVID-19 infection were those related to electrical dysfunction, heart failure, and thromboembolic phenomena. Atrial fibrillation and heart failure stood out due to their high frequency and clinical relevance, with absolute burdens of 10.74 and 11.61 events per 1,000 persons, respectively. A significant increase was also observed in the risk of major adverse cardiovascular events (MACE)—including myocardial infarction, stroke, and death—as well as a general rise in overall cardiovascular disease, with an excess burden of 45.3 additional cases per 1,000 persons.
(13)	To generate coherent evidence on the post-acute sequelae of COVID-19 infection using electronic healthcare records across two regions	Sample: 16,400 patients with a record of COVID-19 infection Follow-up time: 138 to 294 days	Increased risk of vascular and thromboembolic events during the post-acute phase of COVID-19,	HR 1.82 (95% CI: 1.65–2.01) for heart failure HR 1.31 (95% CI: 1.16–1.48) for atrial fibrillation HR 1.32 (95% CI: 1.07–1.63) for coronary artery disease HR 1.74 (95% CI: 1.27–2.37) for deep vein thrombosis HR 2.86 (95% CI: 1.25–6.51) for cardiovascular mortality	A very consistent increase in the incidence of a wide range of long-term sequelae affecting multiple organ systems and all-cause mortality has been observed during the post-acute phase of COVID-19 infection in Hong Kong and the UK.
(21)	To evaluate 1-year cardiovascular outcomes in patients with COVID-19 compared to RT-PCR–negative controls	Sample: 4,427 participants COVID-19 group: 3,578 Control group: 849 Follow-up Time: 1 year	Cardiovascular death, all-cause mortality, venous thromboembolism (VTE), arterial thrombotic events, severe arrhythmias	Cardiovascular death: HR = 1.28 [95% CI: 0.56–2.91]; *p* = 0.555 All-cause mortality: HR = 2.82 [95% CI: 1.99–4.00]; *p* = 0.001 Arterial thrombotic events (ATE): HR = 2.26 [95% CI: 1.02–4.99]; *p* = 0.044 VTE: HR = 9.33 [95% CI: 2.93–29.70]; *p* = 0.001 Severe arrhythmias: HR = 3.37 [95% CI: 1.35–8.46]; *p* = 0.010	Although no significant differences were found in cardiovascular mortality, COVID-19 substantially increased the risk of non-fatal cardiovascular events, particularly venous and arterial thrombosis and arrhythmia. Most events occurred during the acute phase (0–30 days). The post-acute phase showed a residual risk of VTE, particularly deep vein thrombosis.
(24)	To describe the frequency, types, and duration of post-discharge symptoms suggestive of post-COVID syndrome.	Sample: 30 hospitalized patients with COVID-19 discharged between April and December 2020 Follow-up time: 7 to 238 days post-discharge	17 patients (56.7%) developed post-COVID syndrome. Persistent symptoms: cough, fatigue, dyspnea, headache, diarrhea 2 cases with deep vein thrombosis (confirmed by Doppler ultrasound) 1 case of possible reinfection (confirmed by positive RT-PCR after initial recovery) 1 patient with ischemic event (stroke) after discharge Predominantly multisystemic (respiratory, neurological, vascular)	COVID-19 severity and SCA: *p* = 0.029 Lower baseline O₂ saturation in SCA: *p* *=* *0.043*	The study demonstrates a high frequency of persistent post-COVID symptoms among hospitalized patients, with a predominance of respiratory manifestations and severe cases such as thrombosis and stroke. A significant association was identified between the severity of the initial illness and the development of post-COVID syndrome. The absence of multivariable analysis and association measures such as HR or RR limits the causal strength of the findings. Its retrospective design and small sample size restrict the generalizability of the results, although the findings are consistent with similar studies conducted in other regions.
(20)	To assess whether genetic risk (PRS) for VTE is associated with a higher incidence of VTE after COVID-19 vaccination.	Sample: 359,310 vaccinated adults Follow-up time: 28 and 90 days after first and second doses	299 cases of VTE after 90 days of the first dose	PRS was significantly associated with VTE: HR per 1 SD of PRS: • 28 days post-1st dose: HR 1.41 (95% CI 1.15–1.73) • 90 days post-1st dose: HR 1.36 (1.22–1.52) • 28 days post-2nd dose: HR 1.30 (1.04–1.61) • 90 days post-2nd dose: HR 1.33 (1.18–1.49) Consistent with historical unvaccinated cohorts	The study demonstrates that genetic predisposition to VTE, as measured by a polygenic risk score, predicts VTE risk following COVID-19 vaccination with a magnitude comparable to that observed in pre-pandemic periods. This suggests that vaccination does not alter the underlying genetic-VTE association. The finding that the genetic association with VTE is not amplified post-vaccination is critical for public safety, particularly among individuals with inherited thrombophilias.
(26)	To characterise the incidence rates of AESIs following SARS-CoV-2 infection in patients and compare these to historical rates in the general population.	Sample: 23,840,986 patients with COVID-19. Follow-up: 24 months	AESIs: acute myocardial infarction, thrombosis, pulmonary embolism, GBS, encephalomyelitis, among others.	Pulmonary embolism SIR=11.70 (95% CI 10.10–13.70) DVT SIR ≈ 6–9 Acute myocardial infarction SIR ≈ 4.5–6.2 Stroke (nonhemorrhagic) SIR ≈ 2.1–4.5 Guillain-Barré syndrome SIR ≈ 2.8 (95% CI 2.1–3.6) Encephalomyelitis SIR ≈ 1.8 (95% CI 1.05–1.66)	The results indicate that all adverse events of special interest (AESIs) occurred more frequently in patients with a history of COVID-19 compared to the pre-pandemic population. Pulmonary embolism exhibited the highest increase in incidence, occurring more than 11 times as often in post-COVID-19 patients. This finding reflects the hypercoagulable state induced by the virus, particularly in individuals with severe or prolonged illnesses. Other thromboembolic events, such as deep vein thrombosis and acute myocardial infarction, also showed significant increases, further supporting existing evidence that COVID-19 has a substantial impact on the cardiovascular system.
(25)	To analyze the presence of angiographic abnormalities in post-COVID patients exhibiting symptoms indicative of coronary artery disease.	Sample: 40 patients Follow-up: 6 months	Coronary lesions are assessed by angiography.	67.5% (27/40) exhibited angiographic abnormalities. – The most common abnormality was slow coronary flow due to endothelial dysfunction (20%). – Stenosis with thrombosis: • 51%–60% narrowing: 17.5% (7 cases) • 61%–80% narrowing: 7.5% (3 cases) • 81%–90% narrowing: 15% (6 cases) • Total occlusion: 7.5% (3 cases)	The results revealed that 67.5% (27 patients) exhibited some form of coronary abnormality, with the most frequent finding being slow coronary flow due to endothelial dysfunction, followed by varying degrees of stenosis with thrombosis. Although the study does not report association measures such as HR or RR, the findings suggest a strong association between COVID-19 and endothelial dysfunction with thrombotic potential, highlighting a concerning clinical pattern of cardiovascular involvement in the post-infectious phase.
(36)	To characterize VTE in hospitalized patients with COVID-19 and evaluate the safety and efficacy of direct anticoagulants	Sample: 38 patients with VTE among 1583 hospitalized with COVID-19 (2 hospitals); Follow-up time: 25 days	Venous thromboembolism (mainly pulmonary embolism)	Incidence of VTE among COVID-19 patients: 0.82% (38 out of 1,583 patients) Type of thrombotic event: 34 pulmonary embolisms (89%), 3 deep vein thromboses (8%), and 1 pulmonary embolism with concurrent stroke (3%). Initial treatment: 71% received heparin or low molecular weight heparin; 26% were treated with DOACs (apixaban: 8 patients, rivaroxaban: 2 patients); 1 patient received no anticoagulation. Mortality: 9 patients (24%) died.	This observational study suggests that direct oral anticoagulants, such as apixaban and rivaroxaban, may represent an effective and safe alternative to conventional heparin-based therapy in selected patients with COVID-19-associated VTE. The absence of VTE recurrence and bleeding events among patients treated with DOACs, combined with their continued use post-discharge, supports their potential inclusion in prospective clinical trials. However, the small sample size and lack of randomization limit the strength and generalizability of the conclusions.
(30)	To estimate the incidence of post-COVID syndrome and associated symptoms in outpatient care, comparing it with respiratory infections and controls.	Sample: 391,990 COVID-19 patients 62,659 other respiratory infections 659,579 non-COVID controls Follow-up time: up to 2 years (8 quarters)	Medical diagnosis of: Post-COVID Fatigue Chronic fatigue syndrome Dyspnea Psychological disorders Taste/smell disturbances Pulmonary embolism Mild cognitive impairment Myalgia	Chronic Fatigue Syndrome RR (COVID vs. control): ≈ 5.8 RR (COVID vs. RI): ≈ 2.7 Fatigue (symptom) RR (COVID vs. control): ≈ 2.2 RR (COVID vs. RI): ≈ 1.4 Dyspnea RR (COVID vs. control): ≈ 3.07 RR (COVID vs. RI): ≈ 1.94 Smell/Taste Changes RR (COVID vs. control): ≈ 6.36 Highly associated with COVID Pulmonary Embolism Low risk, but higher in COVID. Psychological Disorders RR (COVID vs. control): ≈ 1.57 However, the difference with respiratory infections was not as marked	COVID-19 increases the risk of chronic fatigue syndrome by nearly sixfold. This finding supports the hypothesis that SARS-CoV-2 may trigger persistent inflammatory processes. Taste and smell disorders are among the most distinctive symptoms of COVID-19. The high relative risk suggests neurosensory damage that may persist in the long term. Pulmonary embolism represents a moderate but clinically significant risk, given the potentially fatal nature of the condition and the prothrombotic state induced by COVID-19. Psychological disorders show a notable increase, though less pronounced than physical symptoms. This may be mediated by post-infectious stress or the psychological impact of social isolation and lockdown measures.
(14)	To assess the risks and burden (HR and burden) of incident neurological outcomes at 12 months after COVID-19 infection compared with controls.	Sample: COVID-19: 154,068 Contemporary control: 5,638,795 Historical control: 5,859,621 Follow-up time: 12 months from 30 days post-infection	Incident neurological disorders: cerebrovascular, cognitive, peripheral, mental and sensory	Any neurological disorder HR 1.42 (95%: CI 1.38–1.47) Cerebrovascular disorders HR 1.56 (95%: CI 1.48–1.64) Elevated risk of ischemic stroke (HR 1.50), TIA (HR 1.62), and hemorrhagic stroke (HR 2.19). Cognitive and memory disorders HR 1.80 (95%: CI 1.71–1.88) Includes Alzheimer's disease (HR 2.03) and memory problems (HR 1.77). High risk even in non-hospitalized patients. Peripheral nerve disorders HR 1.34 (95%: CI 1.29–1.39) Peripheral neuropathy (HR 1.34), paresthesias (HR 1.32), dysautonomia (HR 1.30), Bell's palsy (HR 1.48). Extrapyramidal and movement disorders HR 1.42 (95%: CI 1.34–1.50) Mental health disorders HR 1.43 (95% CI 1.38–1.47) Depression (HR 1.44), anxiety (HR 1.38), psychosis (HR 1.51). Importance in post-COVID healthcare planning. Sensory Disorders HR 1.25 (95% CI 1.22–1.28) Loss of smell (HR 4.05), taste (HR 2.26), vision, hearing	The overall risk of developing any neurological disorder increased by 42%. Marked increases were observed in: Cognitive and memory disorders, including Alzheimer's disease. Cerebrovascular events, such as ischemic and hemorrhagic stroke.
(23)	To assess the risk of post-acute complications (persistent symptoms, chronic illness, medication use, and medical care) in individuals with SARS-CoV-2 who did not require hospitalization, compared with SARS-CoV-2-negative individuals	Sample 8,983 SARS-CoV-2 positive (not hospitalized) 80,894 SARS-CoV-2 negative Follow-up time: 2 weeks to 6 months post-test	New prescriptions, new hospital diagnoses, and use of the health system	Bronchodilators (SABA *β*2-agonists) RR 1.32 (95% CI: 1.09–1.60) Increased risk of requiring respiratory therapy Venous thromboembolism RR 1.77 (95% CI: 1.09–2.86) Increased risk of thrombotic events	The study indicates that SARS-CoV-2 positive patients are 32% more likely to initiate SABA therapy compared to negative individuals, suggesting the persistence of respiratory symptoms such as post-COVID dyspnea or cough. Regarding venous thromboembolism, although the absolute risk remains low, the finding is clinically significant. Even mild cases of COVID-19 may predispose individuals to prothrombotic events, supporting hypotheses related to virus-induced systemic inflammation and endothelial dysfuntion.
(27)	To evaluate the incidence of venous VTE, arterial thromboembolic events (ATE) and post-hospital discharge mortality in patients with COVID-19, and the impact of post-discharge thromboprophylaxis	Sample: 4,906 patients hospitalized Follow-up time: Average 92 days post-discharge	Post-discharge events: VTE, ATE, all-cause mortality (MCA), major bleeding	VTE OR: 2.99 (2.00–4.47) if previous VTE. Risk tripled if previous VTE. ATE OR: 2.04 (1.10–3.80) if peripheral arterial disease. Risk doubled. Mortality (MCA) OR: 2.22 (1.78–2.93) if previous ICU stay. High post-discharge mortality in severe cases. Composite event (VTE+ATE+MCA) OR: 3.66 (>75 years); OR: 0.54 with anticoagulation. Advanced age is the greatest risk factor.	7.13% of patients discharged after COVID-19 experienced VTE, TEA, or died within 90 days. This reveals a significant burden of post-acute events. Thromboprophylaxis (rivaroxaban, apixaban, or enoxaparin at prophylactic doses) significantly reduced serious adverse events by nearly 50%, without a proportional increase in the risk of bleeding.
(31)	To evaluate the prevalence and predictors of cardiopulmonary symptoms 90 days after hospital discharge for COVID-19, and their correlation with imaging findings	Sample: 480 patients discharged from hospital due to COVID-19. Follow-up time: 90-day	Present of persistent cardiopulmonary symptoms (dyspnea, cough, fatigue, chest discomfort)	Female sex OR = 3.02 (1.32–6.93), *p* = 0.009 DVT during hospitalization OR = 13.69 (1.07–175.30), *p* = 0.044 Elevated troponin I OR = 1.36 (1.05–1.75), *p* = 0.020 Elevated C-reactive protein OR = 1.06 (1.02–1.10), *p* = 0.001	The study observed that female sex was associated with a threefold increased risk of developing post-COVID symptoms. At 90 days post-discharge, 16.3% of patients hospitalized for COVID-19 exhibited cardiopulmonary symptoms, with fatigue, dyspnea, and chest discomfort being the most frequently reported. Moreover, inflammatory and thrombotic markers during hospitalization including C-reactive protein, troponin I, and a history of deep vein thrombosis (DVT) were significantly associated with the presence of post-COVID symptoms.
(37)	To assess the prevalence and severity of thromboembolic events in people infected with the α, *δ*, and β-micron strains of SARS-CoV-2.	Sample: 3220 patients Follow-up time: 6 months	The common time length to develop and publish COVID-19 thrombotic events for δ 137.18, Omicron 145.18, and α-version turned 149.85 days.	Coronary artery disease 50.00% of all post-COVID-19 thrombotic events. Most frequent vascular complication after infection. Cerebrovascular disease (Stroke) 38.61% of thrombotic events. Significantly associated with older age (*p* < 0.05). Abdominal vascular disease 5.69% of thrombotic events. Similar distribution among SARS-CoV-2 variants. Peripheral arterial disease 5.69% of thrombotic events. No significant difference between variants. Variant distribution (δ, α, Omicron) δ: 14.90%; α: 3.90%; Omicron: 1.68%. Thrombotic events occurred across all variants. Statistically significant difference in mean age (*p* < 0.05). Symptom severity at acute COVID-19 infection 86.17% had moderate to severe symptoms. 13.82% had mild or asymptomatic disease.	Sufferers inflamed with the Delta variant of COVID-19 are greater vulnerable to developing submit COVID-19 thrombotic events with minimum hazard within the Omicron version and intermediate risk within the Alfa version. The hazard of submitting COVID-19 thrombotic activities is directly proportional to the severity of the sickness.
(32)	To present clinical and demographic data of COVID-19 patients, both outpatients and hospitalized, who were monitored for up to 14 months, recording persistent COVID symptoms.	Sample: 646 patients Follow-up time: 14 months	Vascular manifestations	Thrombosis was diagnosed in 20 patients (6.2%)	Older patients tended to have more severe symptoms, leading to a longer post-COVID-19 period. The presence of seven comorbidities was correlated with the severity of infection, and severity itself was the main factor that determined the duration of symptoms in long COVID cases.
(33)	To investigate and characterise the manifestations which appear after eradication of the coronavirus infection and its relation to disease severity.	Sample: 287 patients.	Analysis of post-COVID manifestations revealed that only 10.8% of all subjects have no manifestation after recovery from the disease while a large percentage of subjects suffered from several symptoms.	• Fatigue: 72.8% — most frequent symptom. • Anxiety: 38% • Joint pain: 31.4% • Headache: 28.9% • Chest pain: 28.9% • Dementia and depression: 28.6% each • Dyspnea: 28.2% • Blurred vision: 17.1% • Tinnitus: 16.7% • Intermittent fever: 11.1% • OCD and pulmonary fibrosis: 4.9% each • Diabetes mellitus: 4.2% • Migraine and stroke: 2.8% each • Renal failure and myocarditis: 1.4% each • Arrhythmia: 0.3% — least frequent manifestation	Most of the subjects recovered from COVID-19 experienced several manifestations after the last negative PCR which could be mild symptoms such as fatigue, headache or more critical manifestations like pulmonary fibrosis, stroke and myocarditis.
(35)	To determine radiologic, clinic and laboratory characteristics of COVID-19 positive patients with acute arterial occlusion and compare them with post COVID-19 and non-COVID-19 patients	Sample: 53 patients Follow-up time: 20 months	Lower extremities were most affected, 38 (71.6%), without significant alteration in the coagulogram. Acute arterial occlusion occurred about 2 weeks after the beginning of COVID-19 or at the time of the first appearance of symptoms.	In the group of post COVID-19 patients, thromboembolism occurred after 65 days (in average) with outstanding variability: the earliest incident happened after 19 days from the beginning of the disease and the latest one 180 days after the beginning of COVID-19	Attention should be paid to COVID-19 patients, especially hospitalized in the ICU about 10 days from the onset of the disease in terms of early detection and the treatment of thrombosis and thromboembolism.

COVID-19, Coronavirus Disease 2019; SARS-CoV-2, Severe Acute Respiratory Syndrome Coronavirus 2; PCS, Post-COVID Syndrome; VTE, Venous Thromboembolism; DVT, Deep Vein Thrombosis; PE, Pulmonary Embolism; ATE, Arterial Thrombotic Events; CAD, Coronary Artery Disease; MI, Myocardial Infarction; HF, Heart Failure; ICU, Intensive Care Unit; HR, Hazard Ratio; RR, Relative Risk; OR, Odds Ratio; CI, Confidence Interval; R^2^, Coefficient of Determination; ML, Maximum Lysis; MCF, Maximum Clot Firmness; APTT, Activated Partial Thromboplastin Time; EXTEM, INTEM and APTEM, ROTEM assays assessing coagulation and fibrinolysis; VWF, von Willebrand Factor; VWF(Ag), von Willebrand Factor Antigen; ADAMTS13, A Disintegrin and Metalloproteinase with Thrombospondin Type 1 Motif, Member 13; PRS, Polygenic Risk Score; RT-PCR, Reverse Transcription Polymerase Chain Reaction; CVD, Cardiovascular Disease; DM, Diabetes Mellitus; TIA, Transient Ischemic Attack; SABA, Short-Acting β2-Agonists; MACE, Major Adverse Cardiovascular Events.

#### 3.3.1 Persistence of the procoagulant state and hemostatic dysfunction

The study by Ranucci et al., showed that 97.1% of patients with COVID-19 exhibited a procoagulant pattern during the acute phase, which persisted in 37.3% long-term (up to 18 months). The procoagulant state was maintained in 75% at 3 months, 50% at 6 months, and 25% at 1 year, with a slight increase to 35% at 18 months. Similarly, long-term follow-up in the same study confirmed that patients with significant physical symptoms had a relative risk of 2.8 (95% CI 1.17–6.7; *p* = 0.019) of maintaining hemostatic abnormalities (29). In the research by Constantinescu-Bercu et al., increased thrombogenicity and increased platelet binding and von Willebrand factor (VWF) were found, correlated with elevated levels of VWF Antigen: Disintegrin And Metalloproteinase with Thrombospondin motifs 13 (ADAMTS13) and reduced ADAMTS13 activity (34).

#### 3.3.2 Venous thromboembolism and pulmonary embolism

DVT and PE are among the most frequent and serious complications of post-COVID-19 syndrome. Ortega-Paz et al., reported a significantly elevated risk of PE (HR 5.96, 95% CI 1.85–19.14) and total venous thromboembolism (HR 9.33, 95% CI 2.93–29.70) compared with controls (21). Consistently, Xie et al., estimated, based on a cohort of more than 150,000 individuals, an increased risk for PE (HR 2.93, 95% CI 2.73–3.15), DVT (HR 2.09, 95% CI 1.94–2.24), and superficial venous thrombosis (HR 1.95, 95% CI 1.80–2.12) (22).

Multinational studies confirmed these findings. Voss et al., found a high standardized incidence rate for PE [Standardized Incidence Ratio (SIR) 11.77, 95% CI 10.08–13.73], disseminated intravascular coagulation (SIR 8.79, 95% CI 7.13–10.85), and DVT (SIR 5.56, 95% CI 4.57–6.76), reinforcing the idea of a prolonged systemic thrombotic response following infection (26). Regarding short-term risk, a retrospective study by Junqing Xie et al., identified that the infection was associated with a 21-fold increased risk of venous thromboembolism during the first 30 days (HR 21.42, 95% CI 12.63–36.31), although this risk was reduced in fully vaccinated patients (HR 5.95, 95% CI 1.82–19.5) (20). Roberto et al., demonstrated that persistent cardiopulmonary symptoms were associated with a history of deep vein thrombosis [Odds Ratio (OR) 13.69, 95% CI 1.07–175.30], elevated troponin levels, and depression (OR 6.11, 95% CI 2.25–16.56) (31).

In this group of patients, a relevant clinical recommendation is to initiate anticoagulant therapy at hospital discharge. Kartsios et al. reported that most patients (83%) were discharged on direct oral anticoagulants, with no recorded cases of venous thromboembolism recurrence or post-discharge bleeding events (36).

#### 3.3.3 Arterial thrombotic and ischemic events

Arterial thrombotic events also present as significant sequelae. Lam et al. and Xie et al. showed an increased risk of acute myocardial infarction (HR 1.63, 95% CI 1.51–1.75) and ischemic coronary artery disease (HR 1.66, 95% CI 1.52–1.80) (13, 22). Similarly, Karlovic et al., described cases of acute peripheral arterial occlusion, mainly in the lower extremities, two weeks after the onset of infection (35). Bhandari et al., noted that 7.48% of patients developed post-COVID-19 thrombotic events, primarily coronary artery disease (50%) and cerebrovascular disease (38.6%), with no clear differences between viral variants (37). Kamal et al., also demonstrated that a history of venous thrombosis, elevated D-dimer levels, and C-reactive protein predispose to post-discharge arterial and venous thrombosis (33).

Giannis et al., confirmed that advanced age, a history of venous thromboembolism (OR 2.99, 95% CI 2.00–4.47), intensive care unit stay (OR 2.22, 95% CI 1.78–2.93), and peripheral arterial disease (OR 2.04, 95% CI 1.10–3.80) are the most important risk factors. Furthermore, the use of post-discharge anticoagulation significantly reduced the risk of the combined events (OR 0.54, 95% CI 0.47–0.81) (27). Prolonged cardiovascular effects include endothelial dysfunction and myocarditis. Chhabra et al., found that 95% of patients reported chest pain and 67.5% dyspnea; in addition, coronary slow flow phenomena and intracoronary thrombosis were identified in varying degrees of severity (25).

#### 3.3.4 Cerebrovascular and neurological sequelae

Cerebrovascular events following infection also show a pattern of sustained increase. Xu et al., reported a higher risk of ischemic stroke (HR 1.50, 95% CI 1.41–1.61), transient ischemic attack (HR 1.62, 95% CI 1.50–1.75), and cerebral venous thrombosis (HR 2.69, 95% CI 1.29–5.62) during 12 months of follow-up (14). Lam et al., corroborated the increased risk of stroke (HR 1.20, 95% CI 0.85–1.68) after infection (13), and Rezel-Pots et al., observed a similar, though less pronounced (RR 0.87, 95% CI 0.63–1.11) pattern in a cohort of 13.4 million patients (28).

#### 3.3.5 Systemic and long COVID manifestations

Persistent symptoms following infection are observed in approximately half of patients. De Miranda et al., documented that 50.2% of cases presented with post-COVID-19 syndrome, predominantly fatigue (35.6%), persistent cough (34%), dyspnea (26.5%), and headache (17.3%). Additionally, 6.2% developed thrombosis during follow-up (32). Ogoina et al., reported a high prevalence of persistent symptoms (89.2%), with fatigue being the most frequent (72.8%), accompanied by joint pain, headache, and neuropsychiatric disturbances, while severe manifestations such as stroke, pulmonary fibrosis, and myocarditis were less common (24). Donnachie et al. and Lund et al. complemented these findings by showing a higher frequency of dyspnea [Risk Ratio (RR) 2.00, 95% CI 1.62–2.48], venous thromboembolism (RR 1.77, 95% CI 1.09–2.86) and chronic fatigue (13.3%) compared to control groups (23, 30).

#### 3.3.6 Meta-analysis of events in post-COVID-19 patients

**Figure 2:**
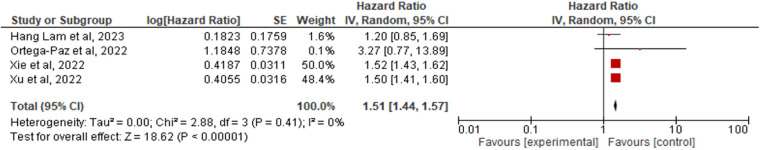
Risk of developing ischemic stroke in post COVID-19 patients.

**Figure 3:**
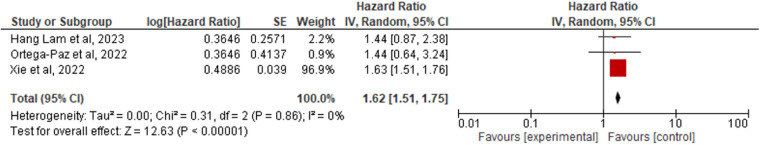
Risk of developing myocardial infarction in post COVID-19 patients.

**Figure 4:**
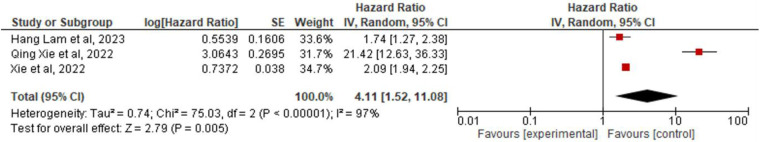
Risk of developing deep vein thrombosis in post COVID-19 patients.

**Figure 5:**
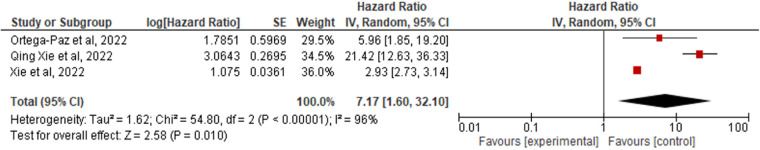
Risk of developing pulmonary embolism in post COVID-19 patients.

The meta-analysis demonstrated a statistically significant association between previous SARS-CoV-2 infection and the subsequent development of major thrombotic events. Using a random-effects model to account for heterogeneity among studies, the pooled hazard ratios indicated an increased risk of IS (HR 1.51, 95% CI 1.44–1.57; *p* < 0.00001) (Figure 2), MI (HR 1.62, 95% CI 1.51–1.75; *p* < 0.00001) (Figure 3), DVT (HR 4.11, 95% CI 1.52–11.08; *p* = 0.05) (Figure 4), and PE (HR 7.17, 95% CI 1.60–32.10; *p* = 0.01) (Figure 5).

## 4 Discussion

This review includes 20 studies reporting thromboembolic events associated with post-COVID-19 syndrome and synthesizes evidence on ischemic stroke, myocardial infarction, deep vein thrombosis, and pulmonary embolism. Overall, the findings indicate an increased risk of thromboembolic events in individuals with post-COVID-19 syndrome, with consistent evidence across study settings suggesting a higher risk of MI.

Patients with post-COVID-19 syndrome exhibited a significant procoagulant state at the time of acute symptoms, in the short term, and throughout long-term follow-up (29). The substantial rise in platelet binding to beta-collagen and anti-von Willibrand Factor A3 associated with post-covid syndrome may provide an explanation (34). Bhandari et al. observed a sequential pattern of thrombotic symptoms, whereby thrombotic events began with coronary artery disorders, followed by cerebrovascular symptoms, abdominal vessels, and peripheral artery diseases (37).

MI is one of the major complications reported in the current literature in post-COVID-19 syndrome patients. Patients with post-COVID-19 syndrome had an increased risk of developing myocardial infarction and ischemic heart disease, according to Rezel-Pots et al. (28). Similar findings are reported in multiple long-term studies compared to the healthy cohort, where our qualitative synthesis also revealed a significant increased risk of MI (13, 20, 21). Additionally, other evident cardiovascular manifestations in this cohort were acute coronary syndrome, ischemic cardiomyopathy, and myocarditis (20, 25, 31, 33). Slow flow due to endothelial dysfunction was a significant finding, which could be the contributing factor (25). A sequential pattern was also seen in this patient population, with thrombotic events starting in the coronary artery, followed by the cerebrovascular, abdominal, and finally peripheral arteries (37).

Several studies reported an increased risk of venous thrombosis, which also aligns with our qualitative data analysis (HR 4.11) (13, 21, 23, 26, 28). According to study results, both short- and long-term follow-up showed increased evidence of venous thrombosis in post-COVID-19 patients (22, 24, 32). Some studies suggested elevated rates of both arterial and venous thrombosis (27). However, Kamal et al. showed a gradual decrease in the incidence of venous thromboembolism, whereas there was no change in the rate of arterial thrombosis over time (33). While multiple treatments were followed based on severity and patient factors, initial treatment started with unfractionated heparin or low molecular weight heparin, progressing to direct anticoagulant and thrombolysis (36, 37). Moreover, a short course of prophylactic or therapeutic anticoagulation after hospital discharge markedly reduced venous thromboembolism (31). Similar to venous thrombosis, our qualitative synthesis showed an increased risk of pulmonary embolism development, which matched the numerous study findings reported in the literature among acute post-covid patients (20, 21, 26, 28).

Compared to the control group, multiple studies found that post-COVID patients had a higher incidence of cerebral vascular events, including transient ischemic attack (TIA) and stroke, which supports our data analysis findings (13, 28, 33, 37). While short-term follow-up showed an elevated incidence rate of ischemic stroke, long-term follow-up resulted in an increased chance of TIA and hemorrhagic stroke (14, 26).

Many recent studies (38–41) and systematic reviews (42–44) give useful insights for comparing the review's results on thromboembolic events after acute COVID-19 syndrome to previous research. Among people recovering from COVID-19, the review emphasizes increased susceptibility to MI, IS, DVT and PE. It also shows that these people have a significant procoagulant condition that persists beyond the acute phase of the illness.

Agarwal et al. conducted a detailed study to explore the broader effects of COVID-19 on cardiovascular and cerebrovascular health, with a focus on thromboembolic events. This research, which included six studies involving more than 12 million patients, found a substantial link between COVID-19 and a higher susceptibility to cardiovascular and cerebrovascular thromboembolic events (45, 46). The discrepancies in incidence rates across populations and healthcare settings underline the necessity of understanding the fundamental causes of these variances.

Another study published in The British Medical Journal investigated the link between COVID-19 vaccinations, thrombocytopenia, and thromboembolic events. This research, which used a self-controlled case-series technique, discovered increased risks of thrombocytopenia and venous thromboembolism after COVID-19 immunization and SARS-CoV-2 infection. These findings suggest that both the virus and the immunization may contribute to blood clots (47).

Ho et al.'s research, also published in The BMJ, investigates the potential hazards of PE, bleeding following COVID-19 infection, and DVT (48). With a substantially higher incidence of PE than DVT, this study suggests that the danger of developing health difficulties persists even after moderate infections. PE has a longer period of increased thromboembolism susceptibility than DVT, up to six months (48, 49). This emphasizes the need for ongoing surveillance and treatment for COVID-19 individuals recovering (48, 50, 51).

Endothelial dysfunction, systemic inflammation, and maybe direct viral invasion of endothelial cells are all thought to be contributing causes to these thromboembolic events. Agarwal et al.'s review looks at potential processes and consequences for public health policy and clinical guidelines. It also encourages further study into molecular insights and new therapeutic options (47, 48).

There are different treatment options for thromboembolic events in post-COVID patients. Some studies recommend a brief course of therapeutic or prophylactic anticoagulation upon hospital discharge to reduce venous thromboembolism (49–51). The evaluation being conducted mentions the use of low-molecular-weight heparin, or unfractionated heparin, which progresses to direct anticoagulants and thrombolysis. Unless contraindicated, the American College of Cardiology recommends pharmacologic venous thromboembolism prophylaxis in all hospitalised patients and provides crucial advice on thromboembolism risk in COVID-19 patients (52–54). The guidance also implies that for hospitalized patients, therapeutic-level anticoagulant doses provide moderate and selective advantages.

### 4.1 Strengths and limitations

Some included studies evaluated thromboembolic outcomes within earlier post-acute periods (<12 weeks). These were retained to capture the full spectrum of post-infectious thrombotic risk and to reflect real-world heterogeneity in follow-up definitions across observational cohorts. Although our methodological framework defined post-COVID according to previously cited criteria (≥3 weeks after acute infection), this variability in outcome timing may have introduced clinical heterogeneity and should be considered when interpreting pooled estimates.

Key strengths of this systematic review include strict adherence to PRISMA guidelines, the use of a comprehensive multi-database search strategy, and systematic methodological appraisal using the NOS. Among the 20 included studies, nine demonstrated high methodological quality with low risk of bias, one showed moderately high quality, nine were classified as moderate quality, and one as low–moderate quality; notably, no studies were categorized as having a high risk of bias. Their inclusion allowed a more complete synthesis of the available evidence on long-term thromboembolic outcomes following COVID-19. Methodologically, the use of dual independent reviewers for screening and data extraction minimized subjectivity and selection bias, while the inclusion of studies from diverse geographic regions enhances the external validity and generalizability of the findings.

This review contributes to the existing body of knowledge by examining the procoagulant condition of post-COVID-19 patients and the associated risks. When integrated with findings from other trials, a complex picture emerges that requires further research to fully understand the long-term cardiovascular consequences of COVID-19. The growing volume of evidence underscores the need for continued and evidence based therapeutic strategies and research focused on post-COVID thromboembolic events.

Nevertheless, several limitations must be acknowledged. Heterogeneity in case definitions for post-COVID-19 syndrome, variation in follow-up periods, and inconsistent reporting of vaccination status and viral variants reduce comparability among studies. The predominance of observational designs, modest sample sizes, and varying outcome definitions introduce potential bias. Incomplete reporting of baseline comorbidities and the limited inclusion of studies from low- and middle-income regions also restrict universal applicability.

Although this review provides a comprehensive synthesis, it leaves a gap in the literature by not directly comparing its findings with those of other studies (55–57). The present work helps address this gap by offering new insights into the risks, causes, and long-term consequences of thromboembolic events following COVID-19 (58, 59). It also emphasizes the importance of individualized treatment strategies based on patient characteristics and disease severity. Despite their heterogeneity, recent studies on thromboembolic events after COVID-19 remain relatively modest in scope.

## 5 Conclusions

This systematic review and meta-analysis provide valuable insights into thromboembolic events among patients with post-COVID syndrome. The findings support an association between post-COVID syndrome and an increased risk of arterial and venous thromboembolic events, including ATE, PE, and DVT, beyond the acute infection period. Despite heterogeneity across studies, consistent patterns were observed suggesting elevated thrombotic risk in this population. These findings should be interpreted with caution, as the available evidence is predominantly observational and causality cannot be inferred. From a clinical perspective, careful monitoring, active screening, and preventive strategies may be warranted in selected patients. Future research should focus on clarifying underlying mechanisms and identifying effective risk-reduction strategies to improve patient outcomes and quality of life.

## Data Availability

The original contributions presented in the study are included in the article/Supplementary Material, further inquiries can be directed to the corresponding author/s.
