# Supplementary material for: Associated thromboembolic events to the post COVID syndrome: a systematic review and meta-analysis
Source: Front Cardiovasc Med. 2026 Jun 11;13:1742868. doi: 10.3389/fcvm.2026.1742868 (PMC13294862; doi:10.3389/fcvm.2026.1742868)
Supplement: Supplementary file 1 [file Datasheet1.docx]

**Supplementarty Material 1. Search Strategies for Databases**

- **PubMed / MEDLINE**

(("COVID-19"[MeSH] OR "SARS-CoV-2"[MeSH] OR COVID-19[tiab] OR SARS-CoV-2[tiab] OR coronavirus*[tiab])
 AND
 ("long COVID"[tiab] OR "post-COVID"[tiab] OR "post COVID"[tiab] OR "post-acute COVID"[tiab] OR "post acute COVID"[tiab] OR
 "post-COVID syndrome"[tiab] OR "post COVID syndrome"[tiab] OR "post-acute sequelae"[tiab] OR PCS[tiab] OR "longterm"[tiab]
 OR "long-term"[tiab] OR persistent[tiab] OR sequela*[tiab])
 AND
 (thrombosis[MeSH] OR "Venous Thromboembolism"[MeSH] OR thrombosis[tiab] OR thromboembol*[tiab] OR "venous thromboembol*"[tiab]
 OR VTE[tiab] OR "pulmonary embol*"[tiab] OR PE[tiab] OR "deep vein thromb*"[tiab] OR DVT[tiab] OR coagulopath*[tiab]
 OR hypercoagulab*[tiab] OR thrombotic[tiab] OR thrombogenic[tiab] OR "cardiovascular disease"[tiab] OR CVD[tiab]
 OR "myocardial infarct*"[tiab] OR stroke[tiab] OR "arterial thromb*"[tiab]))

- **Web of Science**

TS=((covid* OR "sars-cov-2" OR coronavirus*) NEAR/3 (long OR "long covid" OR "post-covid" OR "post acute"
 OR "post-acute" OR "post-acute sequelae" OR PCS OR persistent OR sequela*))
AND
TS=(thromb* OR "venous thromboembol*" OR "pulmonary embol*" OR "deep vein thromb*" OR VTE OR DVT
 OR coagulopath* OR hypercoagulab* OR thrombotic OR thrombogenic OR "cardiovascular disease" OR CVD
 OR "myocardial infarct*" OR stroke)

- **Scopus**

TITLE-ABS-KEY( (covid* OR "sars-cov-2" OR coronavirus*)
 AND ("long covid" OR "post-covid" OR "post acute" OR "post-acute" OR "post-acute sequelae" OR PCS OR persistent OR sequela*)
 AND (thromb* OR "venous thromboembol*" OR "pulmonary embol*" OR "deep vein thromb*" OR VTE OR DVT OR coagulopath*
 OR hypercoagulab* OR thrombotic OR thrombogenic OR "cardiovascular disease" OR CVD OR "myocardial infarct*" OR stroke) )

- **Dimensions**

(covid* OR "sars-cov-2") AND ("long covid" OR "post-covid" OR "post-acute")
AND (thromb* OR "venous thromboembolism" OR VTE OR PE OR DVT)

- **Virtual Health Library** /

(covid* OR "SARS-CoV-2" OR coronavirus)
AND ("long COVID" OR "post-COVID" OR "post-acute" OR persist* OR sequela*)
AND (trombosis OR tromboembolismo OR "venous thromboembolism" OR VTE OR "pulmonary embolism"
OR "deep vein thrombosis" OR DVT OR coagulopat* OR trombótico)

- **British Library**

(covid* OR "sars-cov-2" OR coronavirus*)
AND ("long covid" OR "post-covid" OR "post-acute" OR persistent OR sequela*)
AND (thromb* OR "venous thromboembolism" OR "pulmonary embolism" OR "deep vein thrombosis" OR VTE OR DVT OR coagulopath*)

- **Google Scholar**

"long COVID" OR "post-COVID" OR "post-acute" thrombosis OR "venous thromboembolism"
OR "pulmonary embolism" OR "deep vein thrombosis
