# Supplementary material for: Associated thromboembolic events to the post COVID syndrome: a systematic review and meta-analysis
Source: Front Cardiovasc Med. 2026 Jun 11;13:1742868. doi: 10.3389/fcvm.2026.1742868 (PMC13294862; doi:10.3389/fcvm.2026.1742868)
Supplement: Supplementary file 2 [file Datasheet2.docx]

# Supplementary Material 2. GRADE Summary

| **Study (Author, Year)** | **Certainty Level** |
| --- | --- |
| Ranucci et al. | ⬤⬤◯◯ (Moderate) |
| Rezel-Pots et al. | ⬤⬤◯◯ (Moderate) |
| Constantinescu-Bercu et al. | ⬤⬤◯◯ (Moderate) |
| de Miranda et al. | ⬤⬤◯◯ (Moderate) |
| Xie et al. | ⬤⬤⬤⬤ (High) |
| Lam et al. | ⬤⬤◯◯ (Moderate) |
| Ortega-Paz et al. | ⬤⬤◯◯ (Moderate) |
| Ogoina et al. | ⬤⬤◯◯ (Moderate) |
| Karlovic et al. | ⬤⬤◯◯ (Moderate) |
| Voss et al. | ⬤⬤⬤⬤ (High) |
| Chhabra et al. | ⬤⬤◯◯ (Moderate) |
| Kartsios et al. | ⬤⬤◯◯ (Moderate) |
| Kamal et al. | ⬤⬤◯◯ (Moderate) |
| Donnachie et al. | ⬤⬤⬤⬤ (High) |
| Xu et al. | ⬤⬤⬤⬤ (High) |
| Lund et al. | ⬤⬤⬤⬤ (High) |
| Bhandari et al. | ⬤⬤◯◯ (Moderate) |
| Giannis et al. | ⬤⬤◯◯ (Moderate) |
| Roberto et al. | ⬤⬤◯◯ (Moderate) |
| Junqing Xie et al. | ⬤⬤◯◯ (Moderate) |
